# Analysis of substrate specificity and cyclin Y binding of PCTAIRE-1 kinase

**DOI:** 10.1016/j.cellsig.2012.06.018

**Published:** 2012-11

**Authors:** Saifeldin N. Shehata, Roger W. Hunter, Eriko Ohta, Mark W. Peggie, Hua Jane Lou, Frank Sicheri, Elton Zeqiraj, Benjamin E. Turk, Kei Sakamoto

**Affiliations:** a MRC Protein Phosphorylation Unit, College of Life Sciences, University of Dundee, Dow Street, Dundee DD1 5EH, UK; b Yale University School of Medicine, Department of Pharmacology, 333 Cedar Street, New Haven, CT 06520, USA; c Samuel Lunenfeld Research Institute, Mount Sinai Hospital, 600 University Avenue, Toronto, Ontario, Canada M5G 1X5

**Keywords:** CDK, cyclin-dependent protein kinase, NSF, N-ethylmaleimide-sensitive factor, GST, glutathione S-transferase, HA, haemagglutinin, MBP, myelin basic protein, PSPL, positional scanning peptide library, Cyclin-dependent kinase, CDK16, PCTK1, Cell cycle, Positional scanning peptide library, Proline-directed kinase

## Abstract

PCTAIRE-1 (cyclin-dependent kinase [CDK] 16) is a highly conserved serine/threonine kinase that belongs to the CDK family of protein kinases. Little is known regarding PCTAIRE-1 regulation and function and no robust assay exists to assess PCTAIRE-1 activity mainly due to a lack of information regarding its preferred consensus motif and the lack of *bona fide* substrates. We used positional scanning peptide library technology and identified the substrate-specificity requirements of PCTAIRE-1 and subsequently elaborated a peptide substrate termed PCTAIRE-tide. Recombinant PCTAIRE-1 displayed vastly improved enzyme kinetics on PCTAIRE-tide compared to a widely used generic CDK substrate peptide. PCTAIRE-tide also greatly improved detection of endogenous PCTAIRE-1 activity. Similar to other CDKs, PCTAIRE-1 requires a proline residue immediately C-terminal to the phosphoacceptor site (+ 1) for optimal activity. PCTAIRE-1 has a unique preference for a basic residue at + 4, but not at + 3 position (a key characteristic for CDKs). We also demonstrate that PCTAIRE-1 binds to a novel cyclin family member, cyclin Y, which increased PCTAIRE-1 activity towards PCTAIRE-tide > 100-fold. We hypothesised that cyclin Y binds and activates PCTAIRE-1 in a way similar to which cyclin A2 binds and activates CDK2. Point mutants of cyclin Y predicted to disrupt PCTAIRE-1-cyclin Y binding severely prevented complex formation and activation of PCTAIRE-1. We have identified PCTAIRE-tide as a powerful tool to study the regulation of PCTAIRE-1. Our understanding of the molecular interaction between PCTAIRE-1 and cyclin Y further facilitates future investigation of the functions of PCTAIRE-1 kinase.

## Highlights

► Substrate-specificity requirements of PCTAIRE-1 kinase identified. ► PCTAIRE-tide substrate greatly improved detection of endogenous PCTAIRE-1 activity. ► Binding of PCTAIRE-1 to cyclin Y increases (> 100-fold) PCTAIRE-1 activity. ► Cyclin Y may bind PCTAIRE-1 in a similar way to which cyclin A2 binds CDK2.

## 1 Introduction

The human *CDK16* gene, which encodes PCTAIRE-1, maps to the X chromosome (Xp11.3–p11.23), a chromosomal region associated with a growing number of diseases including neurodegenerative disorders with a genetic basis [1]. PCTAIRE-1 belongs to the CMGC protein kinase family [2], which includes Cyclin-dependent kinases (CDK), Mitogen-activated protein kinases, Glycogen synthase kinase and CDK-like kinases, and is one of three members of the PCTAIRE family (namely PCTAIRE-1, -2 and ‐3). PCTAIRE proteins comprise three principal domains: a central kinase domain that is highly conserved between the three isoforms (~ 80% protein sequence identity), flanked by a long N-terminus and a short C-terminus that are unique for each isoform [3]. The kinase domain of PCTAIREs is closely related to that of other conventional CDKs (e.g. PCTAIRE-1 shares 53.5% sequence identity with CDK2). The CDK family members contain a highly conserved PSTAIRE motif, which plays a key role in cyclin binding, and the interaction between CDKs and cyclins is essential for maximum CDK catalytic activity [4]. As PCTAIREs have retained this important protein-protein interaction motif (albeit the serine residue has been exchanged for cysteine) they are classified into a subgroup of the CDK family, which includes mammalian PFTAIRE, PCTAIRE, PITSLRE, PISSLRE, and more [2].

Because of the well conserved primary sequence between PCTAIRE-1 and CDKs and established roles of CDKs in cell cycle regulation, it was originally hypothesised that PCTAIRE-1 might also be involved in cell cycle control. In support of this, one study demonstrated that endogenous PCTAIRE-1 activity was low during G1 and G1-S phases of the cell cycle, but increased during S and G2 phases [5]. In contrast, other studies showed that PCTAIRE-1 function was not associated with the cell cycle (reviewed in [3]). For example, overexpression of PCTAIRE-1 in neuroblastoma cells had no effect on their cell cycle progression [6]. Therefore, any involvement of PCTAIRE-1 in cell cycle regulation is debatable based on current evidence. Moreover, it is acknowledged that PCTAIRE-1 is expressed in post-mitotic cells and displays a wide tissue distribution with greatest abundance in brain and testis [7,8]. This suggests that its function is not restricted, if at all involved, with the regulation of proliferation/cell cycle. In line with this notion, PCTAIRE-1 has been variously associated with other functions such as, vesicle trafficking [9–11], neurite outgrowth [6], and spermatogenesis [12].

Although PCTAIRE-1 is implicated in a variety of cellular processes, knowledge about its regulation remains elusive. No *bona fide* substrates for PCTAIRE-1 have been identified, and the phosphorylation consensus sequence is unknown, severely limiting current methods to measure PCTAIRE-1 activity. Currently, recombinant and endogenous PCTAIRE-1 isolated from cell/tissue extracts are assayed using generic kinase substrates such as Myelin Basic Protein (MBP) and histone H1 [5–7,10,13]. However, the use of such generic substrates often causes major problems in detecting activity of a particular kinase from cell/tissue extracts by immunoprecipitation, because of confounding activity from inevitable contaminating kinases which renders data interpretation difficult and uncertain. Several members of the CDK family (e.g. CDK2, CDK5) prefer to phosphorylate serine or threonine residues followed by a proline residue and having a basic residue (K/R/H) at the + 3 position (e.g. S/T-P-X-K/R/H, where X represents any amino acid) [14,15]. Therefore, it is predicted, although not yet tested, that PCTAIRE-1 might have the same substrate requirements. In line with this notion, it has been reported that PCTAIRE-1 phosphorylates N-ethylmaleimide-sensitive factor (NSF, a vesicular transport protein that regulates protein exocytosis) at Ser569, which lies within an optimal consensus sequence for conventional CDKs (S*–P–X–R, * represents the phosphorylated residue and X represents any amino acid) [10].

To gain insights into PCTAIRE-1 substrate specificity, we took advantage of positional scanning peptide library technology [16]. We have identified that PCTAIRE-1 has a unique substrate-preference compared to other CDK members and developed a novel peptide substrate termed “PCTAIRE-tide”. Recombinant PCTAIRE-1 displayed vastly improved enzyme kinetics against PCTAIRE-tide compared to a widely used generic CDK substrate peptide. More importantly, PCTAIRE-tide enabled the unambiguous detection of endogenous PCTAIRE-1 activity. Recent studies have proposed a possible PCTAIRE-1 activating subunit. Ou et al. [17] showed that the *Caenorhabditis elegans* homologue of PCTAIRE-1, PCT1, bound to CYY1, the *C. elegans* homologue of human cyclin Y. Moreover, while our manuscript was in preparation, Mikolcevic et al. have confirmed that mammalian PCTAIRE-1 also interacts with cyclin Y [12]. Here we demonstrate that binding of PCTAIRE-1 to cyclin Y increases (> 100-fold) PCTAIRE-1 activity towards PCTAIRE-tide. We have also shed light onto the molecular basis of PCTAIRE-1–cyclin Y interaction and provide a model by which cyclin Y activates PCTAIRE-1. Our structural analysis and mutagenesis data suggest that cyclin Y binds and activates PCTAIRE-1 in a manner reminiscent of the activating interaction between cyclin A2 and CDK2.

## 2 Materials and methods

### 2.1 Materials

PCTAIRE-1C-16 (sc-174) and PCTAIRE-1G6.1 (sc-53410) antibodies were from Santa Cruz Biotechnology. Cyclin Y antibody was from Proteintech. HA.11 (16B2) antibody was from Covance Research Products. The following antibodies were raised in sheep by the Division of Signal Transduction Therapy (DSTT, University of Dundee) against the indicated immunogens: PCTAIRE-1 (S552C, full length human 6×his-PCTAIRE-1), cyclin Y (S206D, full length human GST-cyclin Y). rCDK2–cyclin A2 was prepared by overexpression in *Escherichia coli* as previously described [18]. Peptide substrates for kinase assays were synthesised by GL Biochem, and the peptide library was synthesised by Anaspec, Inc. [γ-^32^P] ATP was from PerkinElmer. HRP-conjugated secondary antibodies were from Jackson Immunoresearch. Unless otherwise indicated all other reagents were from Sigma.

### 2.2 Cloning and mutagenesis

All plasmid constructs were generated and amplified using standard molecular biology techniques. PCTAIRE-1 (NCBI reference NP_006192.1) was amplified from IMAGE EST 3504276 using KOD Hot Start DNA Polymerase (Novagen). The resulting PCR fragment was cloned into pSC-b (Agilent) and sequence verified. The insert was excised using *Not*1 and ligated into the same site in pEBG6P and pCMVFLAG-2. Cyclin Y (NP_659449.3) was amplified from IMAGE EST 6340750 as described above and then cloned into the *Bam*HI and *Not*1 sites in pCMVHA-1. All mutageneses were carried out following the Quick Change method (Agilent) but using KOD Hot Start DNA Polymerase. Cloning of Nuak1 was described previously [19].

### 2.3 Cell culture, transfection and harvesting

Human embryonic kidney (HEK) 293 cells were maintained in high glucose DMEM supplemented with 10% (v/v) foetal bovine serum under 5% CO_2_. Cells were transfected with DNA using polyethylenimine [20] and harvested 36 h post-transfection. Cells were washed with ice-cold PBS and scraped into 50 mM Tris–HCl pH 7.5, 1 mM EDTA, 1 mM EGTA, 0.27 M sucrose, 1% (w/v) Triton X-100, 50 mM NaF, 5 mM Na_4_P_2_O_7_, 1 mM Na_3_VO_4_, 1 mM dithiothreitol (DTT), 1 mM benzamidine and 0.5 mM PMSF. Lysates were clarified at 20,000*g* for 10 min at 4 °C and stored at − 80 °C. Protein concentration was determined using Bradford reagent and BSA standard.

### 2.4 Preparation of GST–PCTAIRE-1

HEK293 cells were transfected with pEBG6P encoding GST-tagged human PCTAIRE-1 and harvested 36 h later as described above. GST–PCTAIRE-1 was isolated by batch-wise purification using Glutathione-Sepharose 4B. Briefly, lysates were incubated with equilibrated resin for 1 h at 4 °C, washed extensively with lysis buffer and eluted with 20 mM reduced glutathione in 50 mM Tris–HCl pH 8, 0.27 M sucrose, 0.1 mM EGTA and 1 mM DTT. Protein concentration was estimated by densitometry of Coomassie-stained gels using BSA standards. PCTAIRE-1 preparations were snap-frozen in N_2(l)_ and stored at − 80 °C.

### 2.5 Positional scanning peptide library screen (PSPL)

**Fig. 1:**
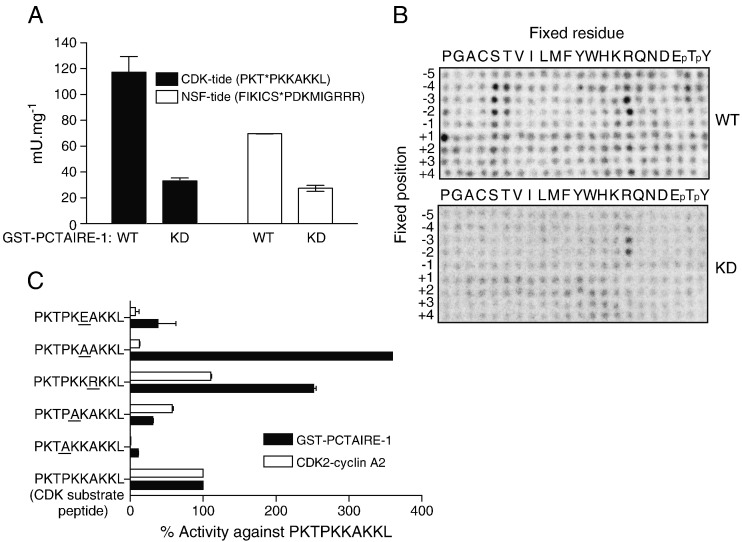
Determination of PCTAIRE-1 optimal substrate motif. (A) GST–PCTAIRE-1 WT and kinase dead (KD, D304A) were prepared by overexpression in HEK293 and 1 μg assayed *in vitro* for phosphotransferase activity against 250 μM of the canonical CDK substrate (CDK-tide, PKT*PKKAKKL) or a peptide derived from the putative PCTAIRE-1 substrate, NSF (NSF-tide, FIKICS*PDKMIGRRR) as described in Materials and methods. (B) GST–PCTAIRE-1 WT/KD was prepared as described in (A) and the optimal substrate motif determined by positional scanning peptide library screening (PSPL) as described in Materials and methods. (C) The activity of GST–PCTAIRE-1 (1 μg) and CDK2–cyclin A2 (10 ng) was assayed against a panel of CDK-tide derivatives (concentration fixed at 250 μM) based on the results of the degenerate library screen (modifications are underlined). Results are expressed as mean ± SEM and are representative of 3 independent experiments.

PSPL screening was performed as previously described [21]. Briefly, peptides had the sequence Y-A-X-X-X-X-X-S/T-X-X-X-X-A-G-K-K(biotin), where X is generally an equimolar mixture of the 17 amino acid residues excluding Cys, Ser and Thr. For each peptide in the set, a single X residue was fixed as one of the 20 unmodified amino acids, phosphothreonine or phosphotyrosine. Aliquots of peptides (0.6 mM) were transferred from a 1536-well stock plate to a 1536-well reaction plate containing 2 μl reaction buffer (50 mM HEPES, pH 7.4, 1 mM EGTA, 1 mM DTT, 10 mM Mg(OAc)_2_, 0.1% Tween 20) in each well using a pin tool equipped with 200 nl stainless steel slot pins (V&P Scientific). Kinase (75 μg of purified WT- or KD-GST–PCTAIRE-1 [Fig. 1B] or 3.75 μg of WT- or KD-FLAG–PCTAIRE-1–cyclin Y complex [Fig. 4C]), rabbit protein kinase inhibitor (PKI, 0.5 μM) and radiolabelled ATP (50 μM, 0.33 μCi/μl [γ-^33^P]ATP) were added together to each well using a strip of 200 nl slot pins, and the plate was sealed and incubated at 30 °C for 2 h. Aliquots (200 nl) were then transferred using a pin tool to a streptavidin membrane, which was washed twice with 0.1% SDS in TBS (10 mM Tris, pH 7.5, 140 mM NaCl), twice with 2 M NaCl, and twice with 1% H_3_PO_4_ in 2 M NaCl. The membrane was then air dried and exposed to a phosphor storage screen to visualise radiolabel incorporation.

### 2.6 Preparation of tissue lysates

Animal studies were approved by the University of Dundee Ethics Committee and performed under a UK Home Office project license. C57BL/6J mice were obtained from Harlan (Leicestershire, UK). Mice were killed by cervical dislocation and tissues rapidly dissected and frozen in N_2(l)_. Tissues were homogenised using a rotor-stator homogeniser (Polytron, Kinematica AG) in lysis buffer, clarified at 13,000*g* for 10 min at 4 °C and stored at − 80 °C. Protein concentration was determined using Bradford reagent and BSA as a standard.

### 2.7 Immunoprecipitation

Cell/tissue lysates were incubated with 1–2 μg antibody (anti-PCTAIRE-1 G6.1 or anti-cyclin Y S206D) and 5 μl protein G-Sepharose for 1 h at 4 °C. FLAG- and HA-tagged proteins were isolated using 5 μl FLAG M2- or HA-agarose respectively. Immune complexes were pelleted at 500g for 1 min and washed 2 × 0.5 ml lysis buffer + 0.15 M NaCl, 2 × Buffer A (50 mM tris pH 8, 0.1 mM EGTA, 1 mM DTT) and eluted with Laemmli sample buffer for analysis by immunoblotting or assayed directly for kinase activity.

### 2.8 Western blotting

Cell/tissue lysates were denatured in Laemmli buffer, separated by tris-glycine SDS-PAGE and transferred to PVDF membrane. Membranes were blocked for 1 h in 20 mM Tris–HCl (pH 7.6), 137 mM NaCl, 0.1% (v/v) Tween-20 (TBST) containing 5% (w/v) skimmed milk. Membranes were incubated in primary antibody prepared in TBST containing 5% (w/v) BSA overnight at 4 °C. Detection was performed using HRP-conjugated secondary antibodies and enhanced chemiluminescent reagent.

### 2.9 Kinase assay

Purified rPCTAIRE-1, rCDK2–cyclin A2 or immune complexes isolated from cell/tissue lysates were assayed for phosphotransferase activity in a final assay volume of 50 μl containing 50 mM HEPES pH 7.5, 0.1 mM EGTA, 10 mM Mg(OAc)_2_, 0.1 mM [γ-^32^P] ATP (300 CPM pmol^− 1^), 1 mM DTT and the indicated concentrations of peptide substrate. Reactions were incubated at 30 °C and terminated by spotting onto P81 paper and immersion in 75 mM H_3_PO_4_. P81 filters were washed 3 × 10 min with H_3_PO_4_, rinsed with acetone, air-dried and incorporation of [γ-^32^P] determined by Cherenkov counting. Results are expressed in U mg^− 1^, where 1 U is defined as the incorporation of 1 nmol phosphate.min^− 1^.

## 3 Results

### 3.1 Purified PCTAIRE-1 poorly phosphorylates the generic CDK substrate peptide

PCTAIRE isoforms are closely related in primary sequence to other members of the cyclin-dependent kinase (CDK) family, including the most widely studied, CDK2. Since many of the CDK family members (e.g. CDK2) prefer to phosphorylate proline-directed serine/threonine residues followed by a basic residue at the + 3 position (consensus S/T*–P–X–K/R [S/T* represents the phosphorylated residue and X represents any amino acid]), we speculated that PCTAIRE-1 might exhibit similar properties. To test this, we assayed the kinase activity of purified GST-tagged PCTAIRE-1 wild-type (WT) and a control kinase-dead/inactive (KD) mutant (in which Asp304 in the Mg^2 +^ binding DFG-motif was substituted to Ala [D304A]), using two peptides conforming to the CDK consensus motif. One substrate has an amino acid sequence of PKT*PKKAKKL, known as CDK substrate peptide, as it was originally derived from CDK *in vitro* phosphorylation sites of histone H1. The other, termed “NSF-tide”, has an amino acid sequence of FIKICS*PDKMIGRRR derived from the surrounding sequence of the reported PCTAIRE-1 phosphorylation site (Ser569) [10] from NSF protein (residues 564 to 575 of human NSF, with an additional three Arg residues at the C-terminal to ensure proper binding to P81 paper). As shown in Fig. 1A, we observed that when using near saturating concentrations (250 μM) of both substrates, PCTAIRE-1 WT showed similar activity towards these two substrates, and this kinase activity was significantly higher than that measured from PCTAIRE-1 KD. However, the specific activity towards CDK substrate peptide by PCTAIRE-1 was vastly lower (> 2000-fold, data not shown) compared to that produced by CDK2–cyclin A2 (expressed and purified from *E. coli*). It could be argued that PCTAIRE-1 isolated from HEK293 cells had only partial activity (possibly due to the absence of activating and/or presence of inhibitory factors), or that the substrate sequences were suboptimal for PCTAIRE-1 activity.

### 3.2 Analysis of substrate specificity of PCTAIRE-1 by a positional scanning peptide library screening (PSPL) approach

Since the optimal phosphorylation consensus for PCTAIRE-1 has not been determined, we have utilised a PSPL [22] to identify an optimal PCTAIRE-1 phospho-peptide. This assay utilised an arrayed library of 198 biotinylated peptide mixtures in which each amino acid residue is systematically substituted at each of 9 positions within the peptide sequence. Recombinant WT-PCTAIRE-1 or KD-PCTAIRE-1, isolated from HEK293 cells, was employed to phosphorylate all 198 peptides simultaneously in solution using radiolabeled ATP, and biotinylated peptides were captured on a streptavidin-coated membrane. Radiolabel incorporation into each peptide was detected by phosphor imaging. As somewhat expected (based on similarity of kinase domain sequences between PCTAIRE-1 and CDK2) the peptide scanning analysis identified that PCTAIRE-1 had a strong preference for proline in the + 1 position relative to the phosphoacceptor site (Fig. 1B). Interestingly, we also observed that PCTAIRE-1 preferentially phosphorylated peptides containing basic residues at the + 2 position (His, Lys or Arg) and + 4 position (His, Lys and Arg), even though signal intensity was modest. We also observed strong signals for peptides with Ser residues fixed at most positions. While this observation is likely to be an artefact arising from the presence of two phosphorylatable sites in those peptides, it does suggest that PCTAIRE-1 is likely to prefer Ser over Thr as the phosphoacceptor residue. In addition, we also observed relatively strong phosphorylation of peptides containing Arg residues at both the ‐2 and ‐3 positions. These Arg signals were also observed in experiments performed with kinase-inactive PCTAIRE-1, indicating that they are likely due to a contaminating kinase activity present in our preparations. Similar results were also reported in previous studies employing recombinant kinase-inactive GST-IκB kinase-β [16] and also kinase-inactive GST-LRRK2 kinase [23] both isolated from HEK293 cells. This trace level of protein kinase activity probably results from kinases present in HEK293 cells that contaminate the GST-tagged protein preparations.

To validate results from the peptide screen, we have obtained a set of synthetic peptides, in which residues from + 1 to + 4 from the phosphoacceptor Thr residue (of the parental CDK substrate peptide) were substituted to various amino acids (mainly to non-charged [Ala] or charged [Arg or Glu] amino acids), and compared the activity of PCTAIRE-1 and CDK2–cyclin A2-complex against these peptides relative to the parental CDK substrate peptide. As predicted, kinase activity of both enzymes was abolished when the Pro residue at the + 1 position was substituted to an Ala (+ 1P → A) (Fig. 1C). Substitution of the Pro residue at the + 1 position with Gly (+ 1P → G) produced similar results (data not shown). When the peptide in which the Lys at the + 2 position was substituted to an Ala (+ 2K → A) was used, activity of both kinases was robustly reduced. Furthermore, PCTAIRE-1 activity was increased ~ 2-fold when the + 4A → R peptide was used, supporting the results obtained from the peptide library screen (Fig. 1B). In contrast, activity of CDK2–cyclin A2 was comparable towards CDK substrate peptide and the + 4A → R peptide. Unexpectedly, PCTAIRE-1 activity showed a marked ~ 3-fold increase when the + 3K → A peptide was used. Coupled to this, was a significant decrease in CDK2–cyclin A2-complex activity to ~ 7-fold lower than its activity on the original CDK substrate peptide when using + 3K → A peptide. Finally, the activity of both kinases against the + 3K → E peptide was negligible. Taken together, these results indicate that PCTAIRE-1 has a strong preference for Pro in the + 1 position relative to the phosphoacceptor site and also prefers a non-charged amino acid (e.g. Ala) at the + 3 position, as both a charged basic (Lys) or an acidic (Glu) residue in that position have a significantly lower kinase activity.

### 3.3 Development of an optimal PCTAIRE-1 substrate

**Fig. 2:**
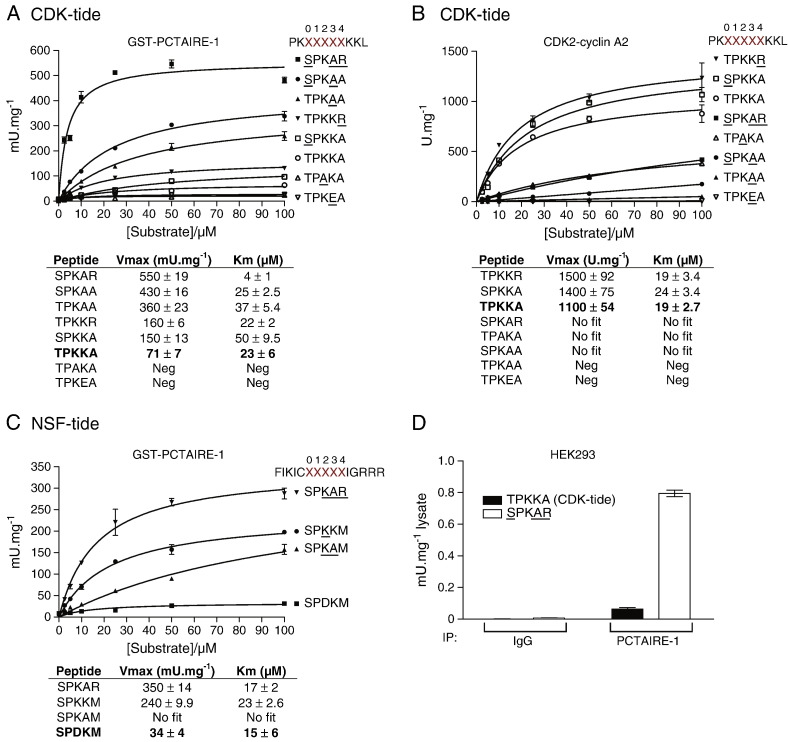
Optimisation of PCTAIRE-1 substrate peptide. The *in vitro* phosphotransferase activity of 1 μg GST–PCTAIRE-1 (A) and 10 ng CDK2–cyclin A2 (B) was determined using a panel of CDK-tide derivatives (modifications from the parental peptide are underlined). Peptide conc. was varied over the range 0 > 100 μM and substrate saturation curves were fitted by non-linear regression to the Michaelis–Menten model. Fitted parameters [*K*_m_ (μM) and *V*_max_ (mU mg^− 1^)] are listed in the adjoining table. (C) Based on the CDK-tide optimisation, derivatives of NSF-tide were synthesised (modifications underlined) and assayed for PCTAIRE-1 activity as described in (A + B). 100% GST–PCTAIRE-1 = 164 mU mg^− 1^ and 100% CDK2–cyclin Y = 940 U mg^− 1^. (D) PCTAIRE-1 was immunoprecipitated from 2 mg of HEK293 lysate and kinase activity determined using 250 μM CDK-tide or the optimised substrate, PKS*PKARKKL (PCTAIRE-tide) as described in Materials and methods. Results are expressed as mean ± SEM and are representative of 3 independent experiments.

We examined additional peptides to consider the potential preference of Ser as phosphoacceptor residue over Thr, a non-charged amino acid (e.g. Ala) at the + 3 position and positive charge at the + 4 position, by steady state kinetic analysis. The most preferred PCTAIRE-1 substrate peptide was PKSPKARKKL (modified from the parental PKTPKKAKKL CDK substrate peptide), which showed a *K*_m_ of ~ 4 μM and *V*_max_ of ~ 550 mU mg^− 1^ (Fig. 2A). This was followed by SPKAA and TPKAA, which also exhibited relatively high substrate affinity (*K*_m_ of ~ 25 μM and ~ 36 μM, respectively). The rest of the peptides, including the parental CDK substrate, showed profoundly lower or negligible affinity for PCTAIRE-1. Regarding the substrate preference for CDK2–cyclin A2, TPKKR, SPKKA and TPKKA peptides showed the highest affinity (*K*_m_ of ~ 20 μM) and *V*_max_ (~ 1100–1500 mU mg^− 1^) (Fig. 2B). The remaining peptides showed profoundly lower or negligible affinity, therefore reliable kinetic values (*K*_m_ and *V*_max_) could not be calculated.

To further elaborate and validate the substrate specificity of PCTAIRE-1, we obtained an additional set of peptides in which residues from + 2 to + 4 of the original NSF-tide (FIKICSPDKMIGRRR) were substituted to various amino acids, and performed kinetic analysis using purified GST–PCTAIRE-1. As shown in Fig. 2C, the affinity of NSF-tide/SPDKM was negligible compared to any of the other peptides tested, and substitution of the aspartic acid at the + 2 position to a basic lysine (SPKKM) significantly increased its affinity (*K*_m_ of ~ 23 μM). This was consistent with the peptide library screen (Fig. 1B) and kinetic analysis using modified CDK substrate peptides (Fig. 2A), and confirmed that PCTAIRE-1 does not preferentially phosphorylate peptides with an acidic residue in the + 2 position from the phosphoacceptor Ser/Thr. It should be noted that contrary to what we observed with the modified CDK substrate peptides, SPKKM had higher affinity than SPKAM, suggesting that surrounding residues are likely to affect substrate affinity. Finally, Fig. 2C shows that, similar to the modified CDK substrate peptides, the modified NSF-tide SPKAR peptide showed highest substrate affinity (*K*_m_ of ~ 17 μM) and *V_max_* (~ 347 mU mg^− 1^). Taken together, we found that a peptide sequence of PKSPKARKKL is the optimal peptide sequence for measuring recombinant PCTAIRE-1 activity *in vitro* and thus we termed it “PCTAIRE-tide” (PCTAIRE kinase-tide).

### 3.4 PCTAIRE-tide substrate enables detection of endogenous PCTAIRE-1 activity

We next wished to validate if the PCTAIRE-tide substrate is an improved tool for measuring endogenous PCTAIRE-1 activity. For this purpose, endogenous PCTAIRE-1 was immunoprecipitated from untransfected HEK293 cell extracts and kinase activity assayed towards both PCTAIRE-tide and CDK substrate peptide. As shown in Fig. 2D, PCTAIRE-1 activity determined using PCTAIRE-tide was significantly higher (~ 10-fold) than using CDK substrate peptide. To rule out the possibility that the obtained kinase activity was derived from non-specific kinase(s) binding to the PCTAIRE-1 antibody (IgG), we used non-specific/generic IgG control, which showed negligible kinase activity (Fig. 2D). A recent study has described that endogenous PCTAIRE-1 activity was undetectable after its immunoprecipitation from HEK293 cell extracts, thus concluded it is “inactive” (data not presented) [12]. It is highly likely that their assays using MBP as substrate were too insensitive to detect PCTAIRE-1 activity reliably.

### 3.5 Tissue expression and activity of PCTAIRE-1

**Fig. 3:**
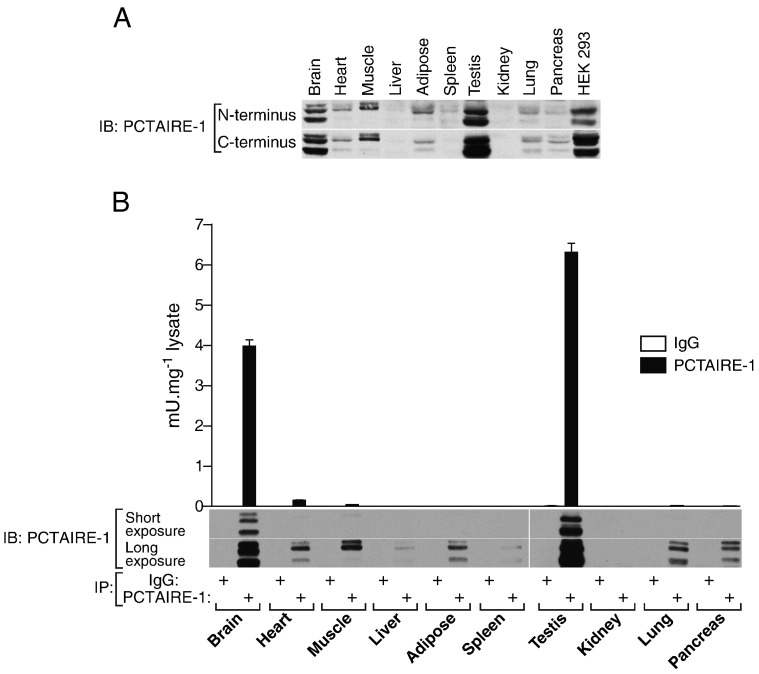
PCTAIRE-1 is most abundant and active in mouse brain and testis. (A) Tissue homogenates were prepared from organs harvested from C57BL/6J mice. Laemmli extracts (40 μg) were separated by SDS-PAGE and immunoblotted using antibodies raised against the N-terminus (G6.1) or C-terminus (C-16) of human PCTAIRE-1. HEK293 was included as a positive control. (B) PCTAIRE-1 was immunoprecipitated from 2 mg of tissue lysate with anti-PCTAIRE G6.1 or rabbit IgG and assayed for kinase activity using 50 μM PCTAIRE-tide. Results are expressed as mean ± SEM and are representative of 3 independent experiments.

After establishing a robust activity assay for PCTAIRE-1, we wished to characterise its expression and activity in different tissues. A panel of mouse tissue lysates as well as extracts from HEK293 cells were immunoblotted using two different PCTAIRE-1 antibodies, one raised against the N-terminal and the other against the C-terminal region of PCTAIRE-1. Fig. 3A confirms previous reports showing that PCTAIRE-1 is highly expressed in brain and testis [7,8]. We also observed that PCTAIRE-1 was expressed at low levels in skeletal muscle, heart, adipose, lung and pancreas extracts, whereas it was not detectable in liver, spleen and kidney extracts. Interestingly, we found that PCTAIRE-1expression is relatively high in HEK293 cells. Two PCTAIRE-1 antibodies produced very similar profiles in terms of tissue distribution, band intensity and pattern of band migration. We next immunoprecipitated endogenous PCTAIRE-1 from the indicated tissue lysates and immunoprecipitants were either used for immunoblotting or assayed for kinase activity using PCTAIRE-tide (Fig. 3B). Generic IgG was used as a negative control. The activity of PCTAIRE-1 was indeed highest in brain and testis [7,12], with no or negligible activity detected from all other tissues (Fig. 3B). The activity data correlated well with immunoblots of precipitated PCTAIRE-1 (Fig. 3B), which corroborated the blots of crude lysate in Fig. 3A. Others have reported that a high-salt wash (0.5 M NaCl) significantly reduced the activity of endogenous PCTAIRE-1 (using MBP as substrate) immunoprecipitated from brain or testis lysate and proposed the existence of a salt-labile factor(s) that influences PCTAIRE-1 activity [7]. However, in our hands, increasing the ionic strength of the wash buffer (0.15 vs. 0.5 M) did not affect PCTAIRE-1 activity when assayed with PCTAIRE-tide (data not shown). It is possible that this “salt-labile factor” was merely a non-specific contaminating kinase (or kinases) removed by a washing buffer containing 0.5 M NaCl.

### 3.6 Cyclin Y binds to PCTAIRE-1 and robustly enhances PCTAIRE-1 activity

**Fig. 4:**
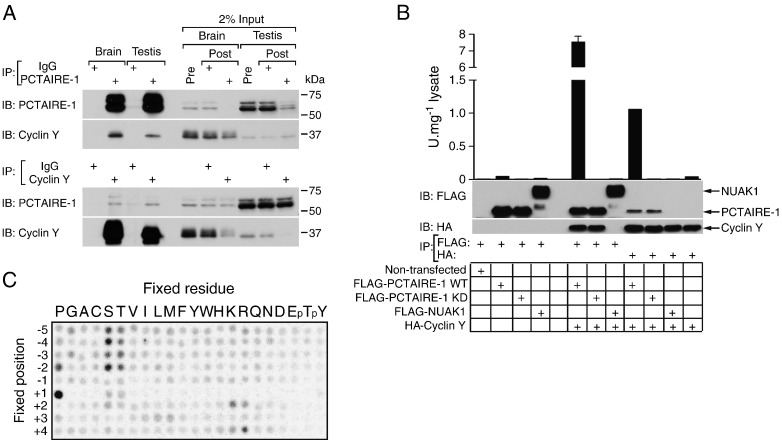
Cyclin Y binds PCTAIRE-1 and robustly enhances kinase activity. (A, upper panel) PCTAIRE-1 was immunoprecipitated from 1 mg mouse brain or testis lysate using anti-PCTAIRE-1 G6.1 or rabbit IgG and immunoblotted for PCTAIRE-1 and cyclin Y. Pre- and post-immunoprecipitation lysate for both tissues (representing 2% total input material) was analysed in parallel to demonstrate the efficiency. (A, lower panel) Alternatively, brain/testis lysate was immunoprecipitated with anti-cyclin Y and blotted as described above. (B) HEK293 were transfected with the indicated pCMV FLAG–PCTAIRE-1 and pCMV HA-cyclin Y constructs. pCMV FLAG-NUAK1 was included as a negative control. Cells were harvested and exogenous PCTAIRE-1/NUAK1 and cyclin Y isolated by immunoprecipitation with FLAG- or HA-agarose respectively. Samples were immunoblotted with the indicated antibodies or assayed for kinase activity using 50 μM PCTAIRE-tide. (C) The PSPL screen was repeated using PCTAIRE-1–cyclin Y complex prepared by co-expression of FLAG–PCTAIRE-1 and HA-cyclin Y in HEK293 and purified using FLAG-agarose. Results are expressed as mean ± SEM and are representative of 3 independent experiments.

Recent studies showed that the *C. elegans* homologue of PCTAIRE-1, PCT1, bound to CYY1, the *C. elegans* homologue of cyclin Y when co-transfected in HEK293 cells [17]. We sought to determine if mammalian endogenous PCTAIRE-1 interacts with cyclin Y. We immunoprecipitated PCTAIRE-1 from brain and testis extracts and the eluted proteins were immunoblotted with either anti-PCTAIRE-1 or anti-cyclin Y antibody (Fig. 4A, upper panel). Indeed, cyclin Y was readily detected in PCTAIRE-1 immunoprecipitates, but not in those precipitated with generic IgG. The reciprocal immunoprecipitation further confirmed this interaction (Fig. 4A, lower panel). It can be noted that immunoblotting of the supernatants (pre- and post-immunoprecipitation) revealed a negligible depletion of cyclin Y after PCTAIRE-1 immunoprecipitation (when PCTAIRE-1 was depleted > 90%) and *vice versa* (Fig. 4A, input lanes), suggesting that only a fraction of cyclin Y proteins bind to PCTAIRE-1 (at least in brain and testis extracts from adult mice). However, we cannot rule out the possibility that cyclin Y has a relatively low affinity for PCTAIRE-1 and the complex dissociates during isolation from the extracts.

Since it has not been determined if catalytic activity is required for PCTAIRE-1 to bind cyclin Y, we next co-expressed FLAG-WT–PCTAIRE-1 or FLAG-KD–PCTAIRE-1 with HA-cyclin Y in HEK293 cells and performed FLAG-/HA-pull-down experiments (Fig. 4B). These experiments clearly verified cyclin Y binds PCTAIRE-1, but not to FLAG-NUAK1 kinase (non-specific control). Notably, cyclin Y binds to both FLAG-WT-PCTAIRE-1 and FLAG-KD–PCTAIRE-1, which demonstrates that catalytic activity is not required for this interaction. Previous studies have shown that cyclin Y is targeted to the plasma membrane by N-terminal myristoylation and therefore an N-terminal epitope tag on cyclin Y might affect efficient or compartmentalised binding [24]. We have performed additional co-immunoprecipitation experiments in HEK293 cells co-transfected with untagged PCTAIRE-1 and untagged cyclin Y and observed similar results (data not shown). Employing PCTAIRE-tide as substrate, a PCTAIRE-1 activity assay was performed on the same samples and showed a vastly increased (> 100-fold) activity when co-expressed with cyclin Y (Fig. 4B). These results suggest that cyclin Y binding to PCTAIRE-1 plays a critical role in regulating PCTAIRE-1 activity.

We repeated the peptide library screen using this significantly more active FLAG- PCTAIRE-1/HA-cyclin Y complex isolated from HEK293 cells. The results (Fig. 4C) identified similar amino acid preferences as in Fig. 1B. Because analysis of the PCTAIRE-1/cyclin Y complex required much smaller amounts of kinase, the contaminating activity observed in our previous analysis with the kinase inactive PCTAIRE-1 was no longer detectable. In addition, analysis of the complex revealed additional selectivity for aliphatic residues at the + 3 position. This additional preference may be a result of an improved signal over background, or alternatively could reflect the influence of the cyclin subunit on peptide phosphorylation specificity. Notably, a similar enhanced preference for aliphatic residues at the + 3 position has been observed for the yeast CDK Pho85 specifically when complexed to the Pho80 cyclin and has been attributed to direct interaction between the peptide and cyclin [25].

### 3.7 Mode of PCTAIRE-1–cyclin Y binding

**Fig. 5:**
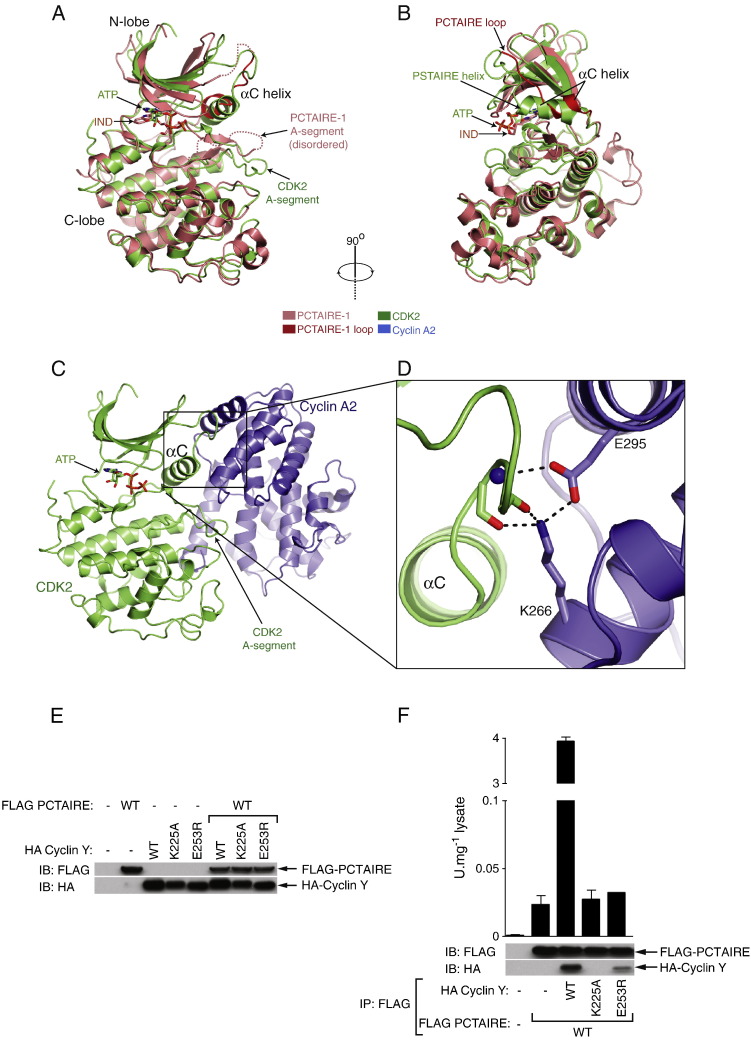
PCTAIRE-1 kinase domain resembles an inactive kinase and binds cyclin Y via a Lys–Glu pair. (A + B) Comparison of the PCTAIRE kinase domain (PDB ID: 3MTL; Structure Genomics Consortium) with the CDK2 structure bound to cyclin A2 (PDB ID: 1FIN; [4]). Helix αC, PCTAIRE loop, PSTAIRE helix and the activation segment (A-segment, defined as the region between DFG and APE motifs) are indicated. IND, indirubin E804. Dashed lines represent disordered regions that are missing from the structure model. (C) Overall structure of CDK2–cyclin A2 complex (PDB ID: 1FIN). (D) Detailed view of cyclin A2 binding to CDK2 through the Lys–Glu pair. Dashed lines represent hydrogen bonds. (E) HEK293 were transfected with the indicated FLAG–PCTAIRE-1 WT and HA-cyclin Y (both WT and mutant) constructs. The resulting lysates were immunoblotted with the indicated antibodies. (F) Lysates from (E) were immunoprecipitated using FLAG-agarose and assayed for kinase activity using 50 μM PCTAIRE-tide. Results are expressed as mean ± SEM and are representative of 3 independent experiments.

To understand the molecular mechanism by which PCTAIRE-1 and cyclin Y interact, we used the crystal structure of CDK2–cyclin A2 complex [4] as a model. Cyclin A2 activates CDK2 by stabilising helix αC and the phosphorylated activation segment that ultimately leads to a productive conformation of a kinase domain [4,26,27]. We analysed the crystal structure of PCTAIRE-1 available from the Protein Databank (PDB ID: 3MTL; Structural Genomics Consortium, June 2010). In contrast to the CDK2 kinase domain, which in the presence of cyclin A2 is in the “closed” active conformation, the PCTAIRE-1 kinase domain adopts an “open” inactive conformation with an unstructured activation segment and αC helix (Fig. 5A and B). This suggests that a binding partner may be required to stabilise the conformation of helix αC and promote a “closed” active PCTAIRE-1 conformation. Since in CDK2 and other CDK family kinases [28–30], this key activatory function is fulfilled by cyclin molecules that bind and stabilise the αC helix and activation segment (Fig. 5C), we hypothesised that cyclin Y binds and activates PCTAIRE-1 in a way similar to which cyclin A2 binds and activates CDK2. To test this hypothesis we used the CDK2–cyclin A2 crystal structure (PDB ID: 1FIN) as a guide to design mutagenesis of interacting residues that would be predicted to disrupt PCTAIRE-1–cyclin Y binding. We looked for cyclin A2 residues that are important for the CDK2–cyclin A2 interaction, and are highly conserved positions between cyclin A2 and cyclin Y (Supplementary Fig. 1). We also ensured that these residues were conserved in cyclin Y genes from different species (Supplementary Fig. 1). Two residues fulfilled our criteria, Lys266 and Glu295 of cyclin A2 (Fig. 5D), which correspond to residues Lys225 and Glu253 of cyclin Y (Supplementary Fig. 1). This Lys–Glu pair forms key hydrogen bonding interactions with backbone atoms of the loop immediately preceding the PSTAIRE helix of CDK2 (Fig. 5D). Importantly, these two residue positions are also conserved in the interactions between CDK2–cyclin B [28], CDK2–cyclin E1 [29], CDK9-cyclin T1 [30], CDK4-cyclin D3 [31,32], CDK6-(viral)-cyclin [33], and Pho85-Pho80 (yeast CDK-cyclin pair; [25]) (Supplementary Fig. 2). The high level of conservation across different CDK and cyclin molecules suggests that the Lys–Glu pair may also fulfil the same role in the PCTAIRE-1–cyclin Y interaction. To test this idea, we mutated the Lys–Glu pair residues and checked if this affected the ability of cyclin Y to bind and activate PCTAIRE-1 (Fig. 5E and F). WT or mutant (K225A or E253R) HA-cyclin Y was transfected alone or co-transfected with WT-FLAG–PCTAIRE-1 in HEK293 cells. As illustrated in Fig. 5E, expression of cyclin Y was comparable to that of WT and expression of PCTAIRE-1 was not affected by co-expression of cyclin Y WT or mutants. We observed that a cyclin Y Lys225Ala mutation completely abolished the PCTAIRE-1–cyclin Y interaction and a Glu253Arg mutation severely affected the ability of cyclin Y to bind PCTAIRE-1 (Fig. 5F). Consistent with the general idea that cyclin binding is required for activation of CDKs, and with our observations that cyclin Y is required for activating PCTAIRE-1 over 100 fold (Fig. 4B), both cyclin Y mutants were incapable of activating the PCTAIRE-1 kinase (Fig. 5F).

## 4 Discussion

By performing positional scanning peptide library analysis, we have revealed key substrate-specificity requirements of PCTAIRE-1 (Figs. 1, 2 and 4C). Although the critical requirement for a proline residue immediately C-terminal to the phosphoacceptor site (+ 1) and preference of a basic residue (His, Lys or Arg) at + 2 are similar to other conventional CDK members, some elements were unique to PCTAIRE. PCTAIRE-1 has a preference for phosphorylating Ser residues followed by a positively charged amino acid (Lys, Arg and His) at the + 4 position. We also found that PCTAIRE-1 prefers amino acids with a small aliphatic side chain (e.g. Ala) rather than charged residues at the + 3 position. These analyses enabled us to generate the PCTAIRE-tide peptide with the consensus sequence S-P-K/R-ϕ-K/R/H (ϕ, small aliphatic amino acid), which we demonstrate is an optimal substrate for measuring PCTAIRE-1 kinase activity. Importantly, PCTAIRE-tide is a robustly improved PCTAIRE-1 substrate when compared to the widely utilised generic protein kinase substrates (MBP and histone H1) or CDK substrate peptide (Figs. 1A and 2A). Currently, a major hurdle in studying PCTAIRE biological functions is the inability to reliably detect specific kinase activity from endogenous PCTAIRE-1 immunoprecipitates. We have overcome this obstacle by using PCTAIRE-tide and were able to assay the activity of endogenous PCTAIRE-1, with minimum or virtually no background activity observed in the control immunoprecipitates (Figs. 2D and 3B). A recent study described that PCTAIRE-1 activity (measured *in vitro* using MBP as a substrate) was not detectable in extracts of HEK293 cells after immunoprecipitation and the authors concluded that PCTAIRE-1 is inactive in this cell line [12]. Consistent with this finding, we observed that PCTAIRE-1 activity towards CDK substrate was marginal and near background levels. By contrast, when PCTAIRE-tide was used, PCTAIRE-1 activity was approximately 20-fold higher than that detected using CDK peptide (Fig. 2D).

It has been reported that NSF can be phosphorylated by PCTAIRE-1 at a proline-directed serine residue on Ser569 in a cell-free assay [10], although it was unknown how efficiently PCTAIRE-1 phosphorylated NSF (as phosphorylation stoichiometry was not reported). Our analysis showed that NSF-tide, developed from the surrounding sequence of Ser569, is a very poor substrate for measuring PCTAIRE-1 kinase activity (Fig. 2C). One of the key reasons for this is that PCTAIRE-1 (and also CDK1/2-cyclin complex [34]) cannot tolerate Asp at + 2 (from Ser569) position (Fig. 2C). Therefore, it is questionable if NSF is a *bona fide* substrate of PCTAIRE-1. To validate NSF as a PCTAIRE-1 substrate, it would be important to observe site-specific phosphorylation (Ser569) of endogenous NSF in intact cells using PCTAIRE-1 knock-down/knock-out cells/tissues with or without an agonist/antagonist (e.g. forskolin treatment which has been shown to inhibit PCTAIRE-1 in HEK293 cells [6,12]).

CDK phosphorylation site preferences have been well established by recent biochemical and structural studies [15,35,36]. The CDKs show a strong preference for substrate peptides that contain a proline at the P + 1 position immediately C-terminus to the phosphorylation residue (known as S/T-P motifs). Given the similarities in primary sequence and the fact that PCTAIRE-1 also prefers a proline at the + 1 position it is possible the molecular basis for this requirement is similar for PCTAIRE-1. However, CDKs can differ widely in conformation (e.g. CDK4 [32]) and substrate specificity is determined by a range of factors including CDK phosphorylation, substrate binding site conformation as well as the type of cyclin bound (i.e. cyclin-mediated substrate recruitment) [15]. To reveal the precise molecular basis for substrate preference of PCTAIRE-1, it is necessary to have a detailed structure from a PCTAIRE-1–cyclin Y complex in the presence of PCTAIRE-tide.

PCTAIRE-1 shares a high percentage identity with other CDK family kinases (e.g. 53.5% identity for PCTAIRE-1 and CDK2 kinase domains) [3]. The crystal structure of PCTAIRE-1 kinase domain is available from the protein databank (PDB ID: 3MTL). PCTAIRE-1 displays the typical kinase fold found in CDK2 and other eukaryotic protein kinases (Fig. 5A). Comparison of PCTAIRE-1 kinase domain structure with the structure of CDK2 bound to cyclin A2 reveals two important differences. The PCTAIRE-1 activation segment is unstructured and differs markedly from the position of the activation segment of CDK2 (Fig. 5A). Furthermore, the PCTAIRE-1 helix αC is different from the so-called PSTAIRE helix of CDK2. In the case of PCTAIRE-1, the region that generally folds into an alpha helix is instead a short helix and consists largely of an unstructured loop (the PCTAIRE loop; Fig. 5B). These analyses suggest that PCTAIRE-1 attains the “open” conformation often seen in protein kinases that are inactive and incapable of phosphorylating substrates [26,27]. By contrast, the structure of CDK2 bound to cyclin A2 shows the “closed” active conformation of CDK2 [4]. This suggests that the PCTAIRE-1 kinase domain alone appears to be in an inactive and unproductive conformation, and cyclin Y may be mimicking the role that cyclin A2 fulfils in activating CDK2. To provide a model by which cyclin Y activates PCTAIRE-1, we performed structural analysis followed by mutagenesis experiments. Here we have identified two cyclin residues (the Lys–Glu pair) that bind backbone atoms from a loop that precedes helix αC of CDKs. The position of this Lys–Glu pair and the interactions these two residues make with CDKs (CDK2, CDK4, CDK6, CDK9 and Pho85), appear to be structurally conserved among five different human cyclins (cyclin 2A, cyclin B1, cyclin D3, cyclin E1, cyclin T1), a yeast cyclin (Pho80) and a viral cyclin (v-cyclin) molecule (Fig. 5C, D and Supplementary Fig. 2). This conservation of the Lys–Glu pair is quite remarkable given the diverse activation mode (at the molecular level) of different CDKs by various cyclins. However, all cyclin molecules share one common mechanism of binding through helix αC and a loop preceding helix αC (Fig. 5C, D and Supplementary Fig. 2). The fact that the Lys–Glu pair makes contacts with backbone atoms rather than specific amino acid side chains of CDKs may have relieved these two residues from evolutionary pressures that have resulted in the many different cyclin-CDK pair interactions. Mutation of the same Lys–Glu pair in cyclin Y also disrupted PCTAIRE-1–cyclin Y interaction, and as expected, these cyclin Y mutants failed to activate PCTAIRE-1 (Fig. 5E and F). Our data suggest that cyclin Y binds and activates PCTAIRE-1 in a manner similar to which cyclin A2 binds and activates CDK2 (Fig. 5). However, it should be noted that there are likely to be unique mechanisms by which PCTAIRE-1–cyclin Y complex formation is controlled, including also the activation segment phosphorylation of PCTAIRE-1. PCTAIRE-1 possesses a unique N-terminal extension (~ 160 residues) and a short C-terminal extension (~ 40–50 residues) prior to and following the kinase domain, respectively [3]. Recently, Mikolcevic et al. showed that PCTAIRE-1 mutants lacking the N-terminal region (∆1–157 or ∆1–121) failed to interact with co-expressed cyclin Y in HEK293 cells [12]. This suggests that, in contrast to other known CDK-cyclin pairs such as CDK2–cyclin A2, the kinase domain of PCTAIRE-1 alone may not be sufficient to bind cyclin Y (at least when co-expressed in cells). Mikolcevic et al. also reported that the phosphorylation of Ser153 on PCTAIRE-1 plays an inhibitory role in binding to cyclin Y. They showed that a Ser153Ala mutant exhibited increased binding to cyclin Y, whereas enhanced Ser153 phosphorylation in response to forskolin treatment (which is known to activate PKA via increasing intracellular cAMP levels) was associated with a decrease in PCTAIRE-1–cyclin Y complex formation in HEK293 cells [12]. Another possibility is that the formation of PCTAIRE-1–cyclin Y complex could be influenced by other previously identified PCTAIRE-1 binding proteins, such as p35 or p11 (reviewed in [3]). In order to form a clear picture of the mechanism by which cyclin Y binds and activates PCTAIRE-1, again, it would be necessary to obtain a crystal structure of PCTAIRE-1–cyclin Y complex.

Analysis of endogenous PCTAIRE-1 kinase activity and PCTAIRE-1–cyclin Y interaction in tissue extracts from adult male C57BL/6J mice revealed that only a small proportion of total cyclin Y binds to PCTAIRE-1 (at least in brain and testis extracts) (Fig. 5A). It is unknown if this is because a major pool of cyclin Y binds to PFTAIRE-1 [24] or other proteins. Alternatively, it is possible that the PCTAIRE-1–cyclin Y complex is formed under particular conditions (during different stages of development, cell cycle, stimulation/inhibition by various factors). We also noted that although endogenous PCTAIRE-1 expression in HEK293 cells was comparable to brain and testis (Fig. 3A), the activity of PCTAIRE-1 was significantly lower in HEK293 cells. This could be possibly due to negligible or non-detectable amount of cyclin Y bound to PCTAIRE-1 in these cells [12].

In conclusion, we have performed biochemical analysis of PCTAIRE-1 substrate specificity and developed a significantly improved assay to assess PCTAIRE-1 activity employing PCTAIRE-tide. PCTAIRE-tide will become a powerful tool in assessing endogenous PCTAIRE-1 in cells/tissues in various biological contexts (e.g. during development, cell cycle), and in response to extracellular agonists, as well as antagonists, which would facilitate understanding the regulation and function of PCTAIRE-1. Moreover, PCTAIRE-tide would be a useful tool to study the regulation and function of PCTAIRE-2 and ‐3 isoforms. It is possible that knowledge of the substrate specificity of PCTAIRE-1 may facilitate the *in-silico* identification of PCTAIRE-1 substrates and/or potential phosphorylation sites within putative substrates. We have also shed light into the molecular basis of PCTAIRE-1–cyclin Y interaction and provide a model by which cyclin Y activates PCTAIRE-1. Our structural analysis and mutagenesis data suggest that cyclin Y binds and activates PCTAIRE-1 in an analogous manner to which cyclin A2 binds and activates CDK2.

## Author contribution

Kei Sakamoto and Saif Shehata planned the experiments and Saif Shehata, Roger Hunter, and Kei Sakamoto analysed the experimental data. Saif Shehata performed most of the experiments shown in Figs. 1–5, while Roger Hunter (Fig. 1–5) and Eriko Ohta (Fig. 3) provided technical/intellectual support and trouble shooting of the experiments. Benjamin Turk designed and Hua Jane Lou performed the positional scanning peptide library analysis (Figs. 1B and 4C) and Benjamin Turk also provided intellectual support in designing experiments and interpreting data shown in Figs. 1C and 2. Elton Zeqiraj performed the structural analysis for Fig. 5 and supplementary material and provided intellectual support in designing experiments and interpreting data shown in Fig. 5. Frank Sicheri supervised Elton Zeqiraj. Kei Sakamoto and Saif Shehata wrote the overall draft and Benjamin Turk, Elton Zeqiraj and Roger Hunter all contributed to improve/edit the final draft.

## Funding

We thank the British Medical Research Council and the pharmaceutical companies supporting the Division of Signal Transduction Therapy Unit (AstraZeneca, Boehringer-Ingelheim, GlaxoSmithKline, Merck & Co. Inc., Merck KGaA and Pfizer) for financial support. E. Z. is supported by a Sir Henry Wellcome Postdoctoral Fellowship. R.W.H is supported by the British Heart Foundation and E.O. is supported by Diabetes UK. This work was supported by National Institutes of Health grant R01 GM079498 (to B.E.T.).
