# Supplementary material for: Analysis of substrate specificity and cyclin Y binding of PCTAIRE-1 kinase
Source: Cell Signal. 2012 Nov;24(11):2085–94. doi: 10.1016/j.cellsig.2012.06.018 (PMC3590450; doi:10.1016/j.cellsig.2012.06.018)
Supplement: Supplementary file 1 — Supplementary materials [file mmc1.pdf]

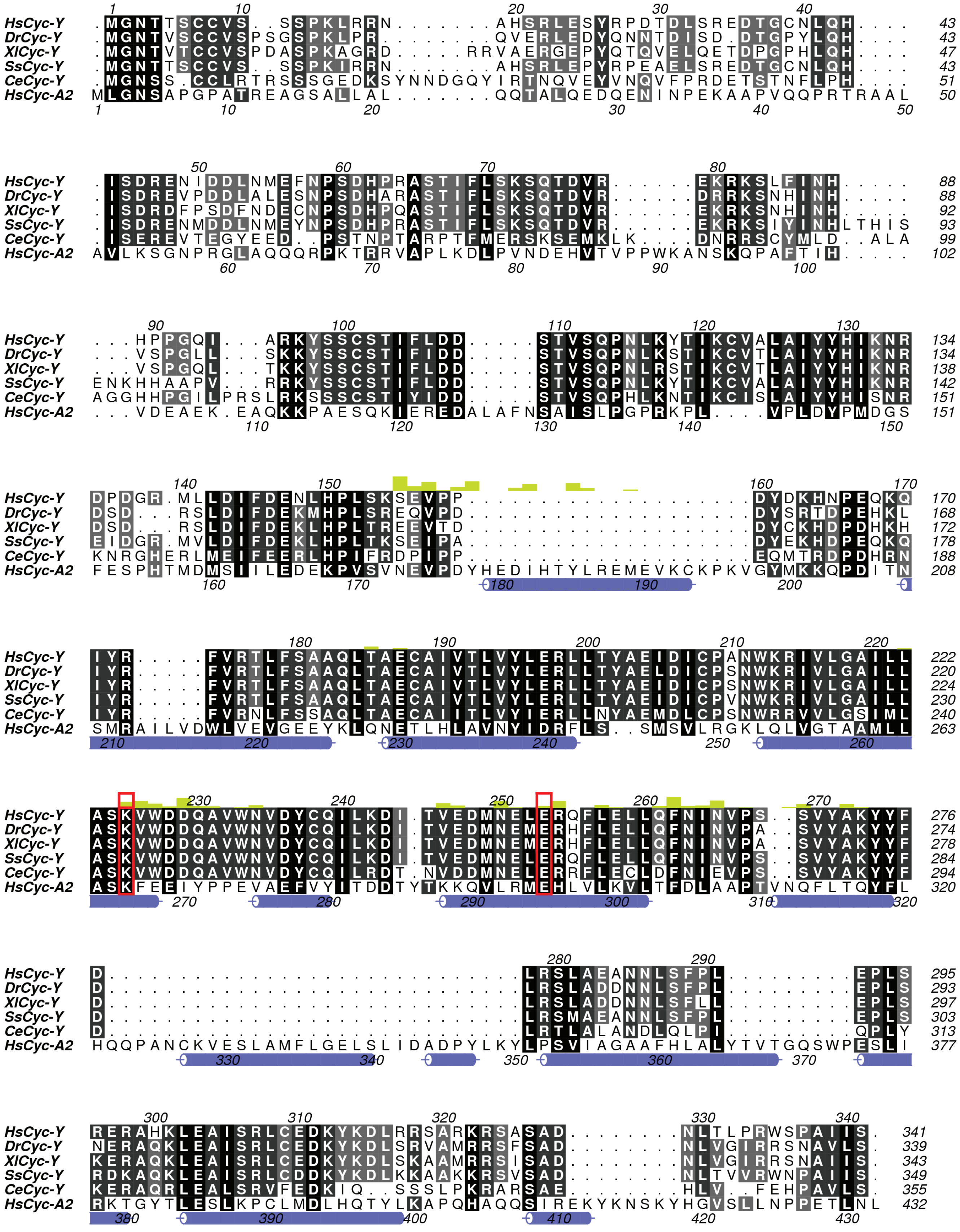

Supplementary figure 1. Sequence conservation analysis of cyclin Y. Multiple sequence alignment (black = conserved, white = not conserved) of cyclin Y and cyclin A2 from the indicated species. (Hs = *H. Sapiens*, Dr = *D. rerio*, Xl = *X. laevis*, Ss = *S. salar*, Ce = *C. elegans*. Alignments were performed with MUSCLE [1] and edited and displayed using the program ALINE [2]. A graph of residues involved in cyclin A2- CDK2 interaction against their contact area (green bars), is displayed. Height of the bar represents the contact area (atom pairs closer than 3.9 Å, analysed by CONTACT from the CCP4 package (Collaborative Computational Project, Number 4, 1994)), divided by the molecular weight of the participating amino acid. The amino acid positions of the Lys-Glu pair are boxed. The secondary structure of cyclin A2 is shown in blue. *H. sapiens* cyclin Y numbering is given at the top and *H. sapiens* cyclin A2 numbering at the bottom.

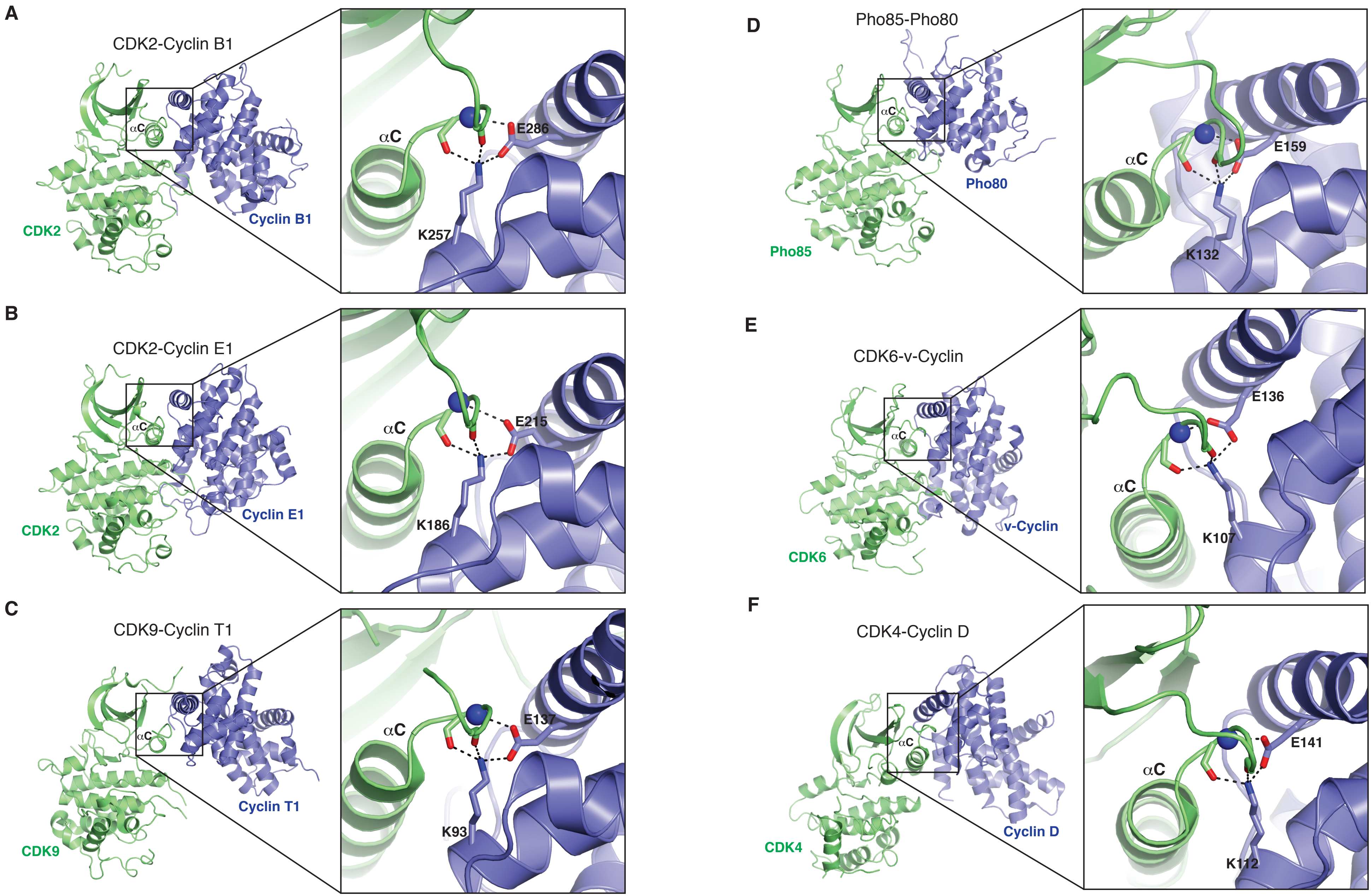
A
CDK2-Cyclin B1
αC
CDK2
Cyclin B1
αC
E286
K257
B
CDK2-Cyclin E1
αC
CDK2
Cyclin E1
αC
E215
K186
C
CDK9-Cyclin T1
αC
CDK9
Cyclin T1
αC
E137
K93
D
Pho85-Pho80
αC
Pho80
Pho85
αC
E159
K132
E
CDK6-v-Cyclin
αC
v-Cyclin
CDK6
E136
αC
K107
F
CDK4-Cyclin D
αC
Cyclin D
CDK4
E141
αC
K112

Supplementary figure 2. Structural conservation and position of the Lys-Glu pair in CDK-cyclin interactions. A) Overall structure of CDK2-cyclin B1 complex (PDBID 2JGZ; [3]. On the right, details of the cyclin B1 Lys-Glu pair interaction with CDK2. B) Overall structure of CDK2-cyclin E1 complex (PDBID 1W98; [4]). On the right, details of the cyclin E1 Lys-Glu pair interaction with CDK2. C) Overall structure of CDK9-cyclin T1 complex (PDBID 3BLH; [5]. On the right, details of the cyclin T1 Lys-Glu pair interaction with CDK9. D) Overall structure of yeast Pho85-Pho80 complex (PDBID 2PMI; [6]. On the right, details of the Pho80 Lys-Glu pair interaction with Pho85. E) Overall structure of CDK6-v-cyclin complex (PDBID 2EUF; [7]). On the right, details of the v-cyclin Lys-Glu pair interaction with CDK6. F) Overall structure of CDK4-cyclin D complex (PDBID 2W9F; [8]). On the right, details of the cyclin D Lys-Glu pair interaction with CDK4.

REFERENCES

1 Edgar, R. C. (2004) MUSCLE: multiple sequence alignment with high accuracy and high throughput. Nucleic Acids Res. **32**, 1792-1797

2 Bond, C. S. and Schuttelkopf, A. W. (2009) ALINE: a WYSIWYG protein-sequence alignment editor for publication-quality alignments. Acta Crystallogr D Biol Crystallogr. **65**, 510-512

3 Brown, N. R., Lowe, E. D., Petri, E., Skamnaki, V., Antrobus, R. and Johnson, L. N. (2007) Cyclin B and cyclin A confer different substrate recognition properties on CDK2. Cell Cycle. **6**, 1350-1359

4 Honda, R., Lowe, E. D., Dubinina, E., Skamnaki, V., Cook, A., Brown, N. R. and Johnson, L. N. (2005) The structure of cyclin E1/CDK2: implications for CDK2 activation and CDK2-independent roles. EMBO J. **24**, 452-463

5 Baumli, S., Lolli, G., Lowe, E. D., Troiani, S., Rusconi, L., Bullock, A. N., Debreczeni, J. E., Knapp, S. and Johnson, L. N. (2008) The structure of P-TEFb (CDK9/cyclin T1), its complex with flavopiridol and regulation by phosphorylation. EMBO J. **27**, 1907-1918

6 Huang, K., Ferrin-O'Connell, I., Zhang, W., Leonard, G. A., O'Shea, E. K. and Quiocho, F. A. (2007) Structure of the Pho85-Pho80 CDK-cyclin complex of the phosphate-responsive signal transduction pathway. Mol Cell. **28**, 614-623

7 Lu, H. and Schulze-Gahmen, U. (2006) Toward understanding the structural basis of cyclin-dependent kinase 6 specific inhibition. J Med Chem. **49**, 3826-3831

8 Day, P. J., Cleasby, A., Tickle, I. J., O'Reilly, M., Coyle, J. E., Holding, F. P., McMenamin, R. L., Yon, J., Chopra, R., Lengauer, C. and Jhoti, H. (2009) Crystal structure of human CDK4 in complex with a D-type cyclin. Proc Natl Acad Sci U S A. **106**, 4166-4170
